# Tuberculosis-like respiratory infection in 245-million-year-old marine reptile suggested by bone pathologies

**DOI:** 10.1098/rsos.180225

**Published:** 2018-06-06

**Authors:** Dawid Surmik, Tomasz Szczygielski, Katarzyna Janiszewska, Bruce M. Rothschild

**Affiliations:** 1 Faculty of Earth Sciences, University of Silesia, Będzińska 60, 41-200 Sosnowiec, Poland; 2 Park of Science and Human Evolution, 1 Maja 10, 46-040 Krasiejów, Poland; 3 Institute of Paleobiology, Polish Academy of Sciences, Twarda 51/55, 00-818 Warsaw, Poland; 4 Carnegie Museum, 4400 Forbes Avenue, Pittsburgh, PA 15213, USA; 5 West Virginia University School of Medicine, Morgantown, WV 26506, USA

**Keywords:** osteopathology, palaeopathology, tuberculosis, Triassic, ecotrophism

## Abstract

An absence of ancient archaeological and palaeontological evidence of pneumonia contrasts with its recognition in the more recent archaeological record. We document an apparent infection-mediated periosteal reaction affecting the dorsal ribs in a Middle Triassic eosauropterygian historically referred to as ‘*Proneusticosaurus*’ *silesiacus*. High-resolution X-ray microtomography and histological studies of the pathologically altered ribs revealed the presence of a continuous solid periosteal reaction with multiple superficial blebs (protrusions) on the visceral surfaces of several ribs. Increased vascularization and uneven lines of arrested growth document that the pathology was the result of a multi-seasonal disease. While visceral surface localization of this periosteal reaction represents the earliest identified evidence for pneumonia, the blebs may have an additional implication: they have only been previously recognized in humans with tuberculosis (TB). Along with this diagnosis is the presence of focal vertebral erosions, parsimoniously compared to vertebral manifestation of TB in humans.

## 1. Introduction

Pathological conditions observed in fossil skeletal remains provide an exceptional opportunity for epidemiologic study of disease. Recognition of pathogens permits investigation of how disease affected ancient biota [1,2]. The fossil record, although very selective and incomplete, allows resolution of gaps in our understanding of their evolution and transmission.

Osseous abnormality/pathology is relatively common in reptiles [3,4]. Most of the conditions described thus far have been identified in terrestrial or semi-terrestrial taxa. They include post-traumatic malformation [5,6], congenital defects [7,8] and neoplasms [3,9–11]. Previous reports of Mesozoic marine reptile pathology have been limited to recognition of bone necrosis, of both aseptic [12] and infectious [13] aetiology. Aseptic avascular necrosis associated with decompression syndrome is quite common in ancient marine reptiles, including sea turtles [14,15], mosasaurs [16], ichthyosaurs [12] and sauropterygians [12,17]. The fossil record of infectious disease in marine reptiles is scarce, limited to bacterial infections in Triassic sauropterygians [13] and Cretaceous mosasaurs [18,19]. Fossilization of actual microorganisms is exceptionally rare (e.g. *Yersinia* strains have been found in Eocene flea) [20].

Pneumonia, the result of infection of lung parenchyma, affects 450 million people yearly [21]. It is one of the oldest diseases known to humankind, described for the first time by Hippocrates [21].

Among the organisms responsible for bacterial pneumonia is the trans-phylogenic zoonosis tuberculosis (TB), caused by *Mycobacterium tuberculosis* and related organisms. This contagious disease has been previously documented in reptiles [2,3,22–24] and marine tetrapods [22,25–27]. It is commonly recognized in the archaeological record [2,28–32] and also documented in Pleistocene bovids [33–35]. Diagnosis of TB, on the basis of osteological data, *M*. *tuberculosis* lipid biomarkers and DNA sequence detection, was established by Hershkovitz *et al*. [36] in Neolithic human bones and by Rothschild *et al*. [33] and Lee *et al*. [37,38] in Pleistocene bison. Vertebral collapse and coalescence, classic complications of TB [2,39], were also documented in a Pliocene macropod (marsupial) [2]. Molecular data suggest that *M*. *tuberculosis* diverged from its common ancestor as recently as 17 000 years ago [33,38], but that perspective is difficult to reconcile with actual recognition of the disease in 17 000 and 75 000–100 000 ypb bison [33]. Thus, the antiquity of TB has not been established.

Herein, we report a TB-like respiratory infection in a Triassic (approx. 245 million ybp) marine reptile. The presumptive diagnosis is established on the basis of macroscopic, radiological and histological study of its rib and vertebrae pathology.

## 2. Methods

### 2.1. Thin-sectioning

The proximal portion of the rib fragment was thin-sectioned and examined using a Nikon Eclipse 80i transmitted light microscope fitted with a DS-5Mc cooled camera head. The distal part of the same specimen, around 0.5 cm long, was subjected to X-ray microtomography (XMT) scanning.

### 2.2. X-ray microtomography

Radiologic evaluation was performed with an Xradia MicroXCT-200 imaging system, equipped with a 90 kV/8 W tungsten X-ray source. The scans were performed using the following parameters: voltage: 50 kV; power: 6 W; exposure time: 4 s; voxel size: 4.61 µm. Images were reconstructed with the XMReconstructor software provided with the Xradia system. Three-dimensional images of bone and XMT sections were obtained by processing with the Avizo 7.0 Fire Edition software.

## 3. Results

**Figure 1.**
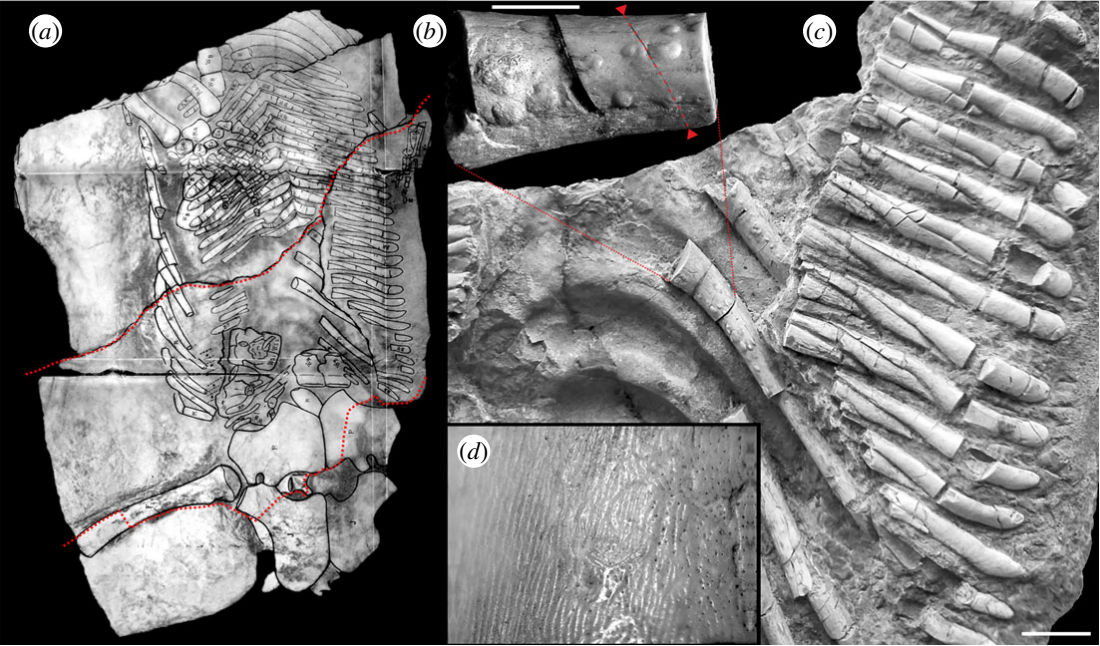
‘*Proneusticosaurus*’ *silesiacus* holotype, MG UWr. 4438s. (*a*) The appearance of the specimen in the ventral view (with bones outlined) before it was damaged during World War II (see the electronic supplementary material). Reproduction from Volz [40], Plate 15 with written permission of Schweizerbart—Publishers (http://www.schweizerbart.de/journals/pala). Dotted line indicates the outline of the remaining middle portion of the specimen. Not to scale. (*b*) Extracted fragment of a dorsal rib used in this study. Arrowheads and the dashed line indicate the point of separation of the proximal part of the specimen (left) used for histological sections, and the distal part (right) used for XMT. Scale bar equals 5 mm. (*c*) Close-up of the specimen, as it appears now, showing dorsal ribs with bleb-like protrusions. Indicated is the position of the extracted rib fragment. Serial elements to the right are gastralia. Scale bar equals 10 mm. (*d*) A fingerprint-like surface of bone and numerous points of entrance for blood vessels, not to scale.

Pneumonia-related bone involvement was recognized on the visceral surface of dorsal ribs of the holotype MG UWr 4438 (figure 1*a*,*c*; electronic supplementary material, figure S1) of the Middle Triassic marine reptile ‘*Proneusticosaurus*’ *silesiacus* Volz [40]. We agree with previous authors [41–43], who interpreted the stated genus ‘*Proneusticosaurus*’ as actually representing the postcranial remains of another taxon, *Cymatosaurus*. The latter, however, is known thus far only from cranial material. Complete description of the specimen, associated vertebral collapse and fusion of vertebral centra (typical of TB-related Pott's disease in humans) and additional illustrations of rib pathology are included in the electronic supplementary material.

**Figure 2.**
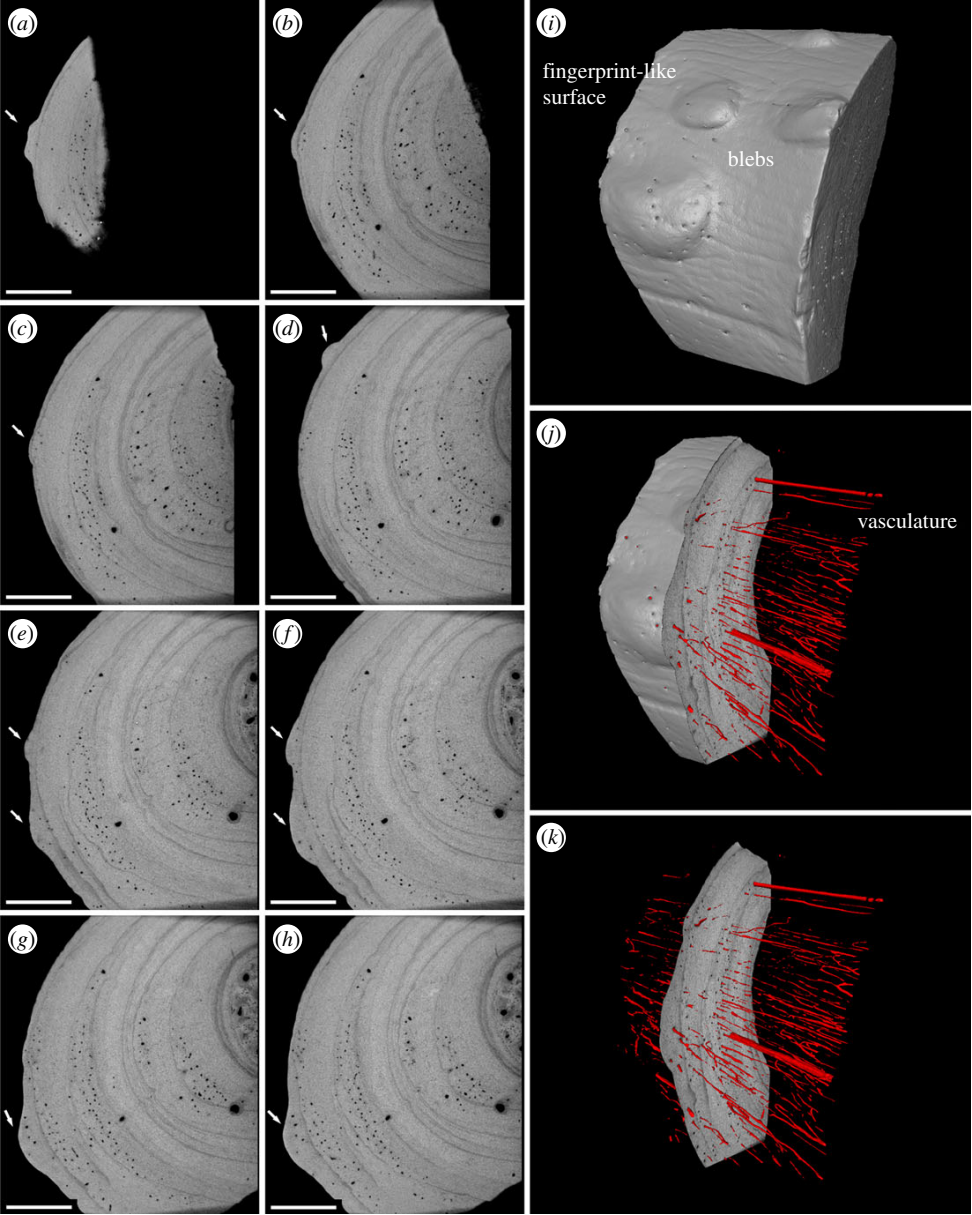
X-ray microtomographic images and three-dimensional reconstruction of the dorsal rib fragment of ‘*Proneusticosaurus*’ *silesiacus* holotype, MG UWr. 4438s. (*a*–*h*) XMT virtual sections (distal towards proximal) of the rib fragment showing superficial blebs (arrows). Note the increased vasculature within the blebs, and the bleb-like shape of older zones. Scale bar equals 1 mm. (*i*–*k*) Three-dimensional visualizations of several blebs on the rib surface as well as bone vasculature in the cropped fragment of cortical bone. Note the presence of a fingerprint-like system of superficial vascular grooves (*i*). (*i*–*k*) Not to scale. Ventral (visceral) towards the left.

Bone reaction (figure 1*b*) is present on the visceral surface of at least four consecutive dorsal ribs on the left side of the animal (figure 1*b*). The lesions are elliptical in shape, ranging in size from 0.5 to 3 mm, and appear contiguous with, rather than applied to the bone surface. XMT revealed bleb-shaped thickening of the periosteal lining of the bone, interrupted internally by multiple branching channels about 50 µm in diameter (figure 2).

The blebs have relatively smooth surfaces and an unbroken outline, although one does have serpentine bifurcating grooves on its surface (figure 1*d*). The grooves most probably represent the imprint of overlying blood vessels, rather than the result of irregular bone deposition. Very similar, but weaker fingerprint-like texture is also present on non-pathological regions of the ribs and was previously reported on the vertebrae and appendicular skeleton of other sauropterygians (e.g. pachypleurosaurids) [44].

**Figure 3.**
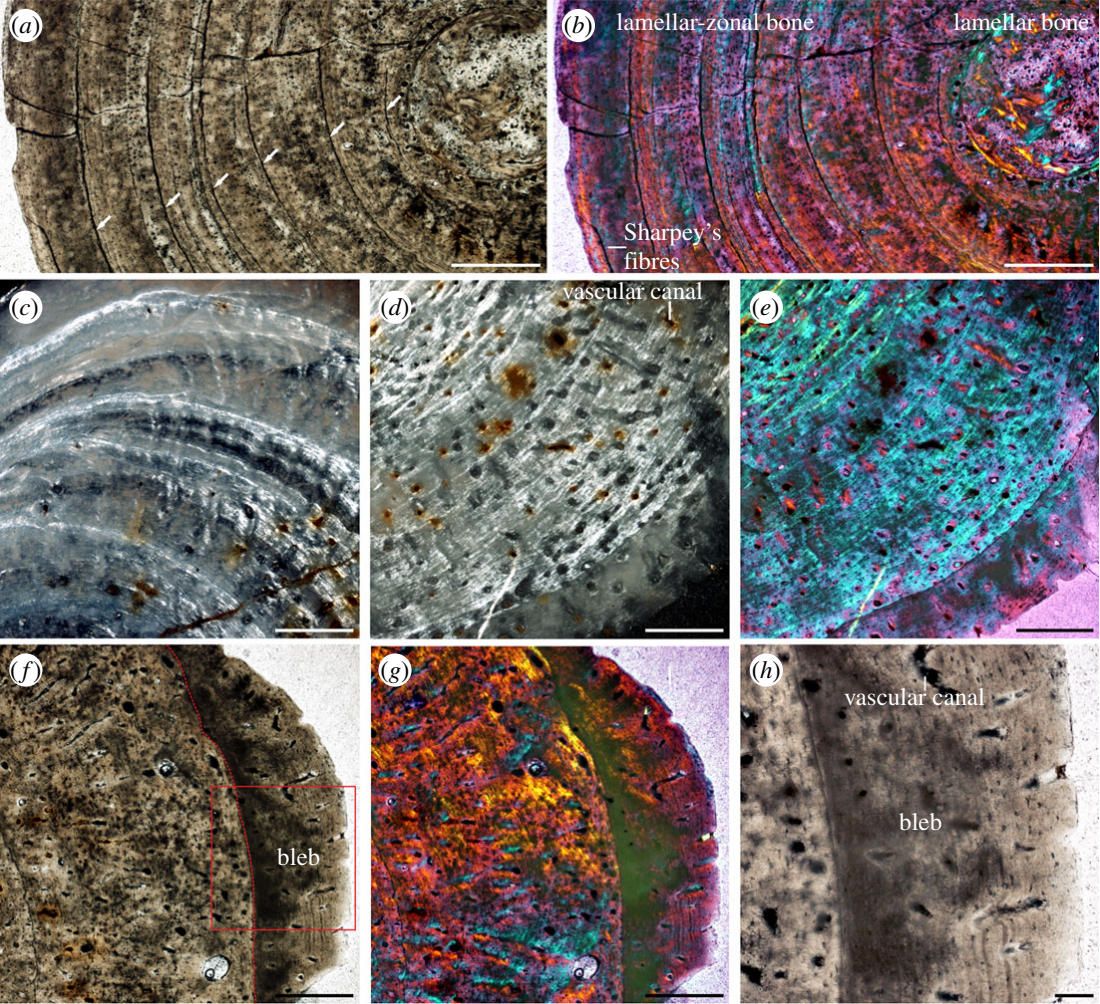
Histology of the dorsal rib of ‘*Proneusticosaurus*’ *silesiacus* holotype, MG UWr. 4438s. (*a,b*) The dorsal region of the rib composed of avascular lamellar-zonal bone with well-pronounced zonation, and the medullar area in transmitted light (*a*) and polarized light with *λ* compensation (*b*). Arrows show LAGs. (*c*) Anterior region of the rib in polarized light exhibiting the vascularization, and rate of bone deposition gradually increasing towards the ventral (visceral) region. Note the wavy organization of the tissue. (*d,e*) Ventro-posterior region of the rib in polarized light without (*d*) and with (*e*) *λ* compensation showing the vasculature increasing even more and attaining radial organization towards the ventrum. (*f,g*) Ventral region of the rib in transmitted (*f*) and polarized light with *λ* compensation (*g*), presenting the radial vasculature and the bleb. Indicated is the LAG separating the pathological outer zone of the bone (dotted line) and the area shown in panel (*h*). (*h*) Close-up of the bleb in transmitted light. Scale bars for panels (*a–g*) equal 0.5 mm, for panel (*h*) equals 0.1 mm. In all panels ventral (visceral) towards the right-hand side.

Histological documentation of penetrating branching channels reveals the infectious nature of the pathologic process. The channels appear limited to the internal aspect of the inflated outer cortex. Underlying bone was otherwise unaffected, except for increased local vascular supply. The ribs are composed of a lamellar-zonal bone tissue, with very minimal remodelling. Zonation is of greatest density and best visualized dorsally (figure 3*a*,*b*), where growth cycles consist primarily of parallel-fibred bone, grading into lamellar bone and culminating in lines of arrested growth (LAGs). Organization of collagen fibre traces in a wavy manner (figure 3*c*) marks external migration of blood vessels during bone growth. Some of the LAG waves appear to represent remnants of superficial vascular grooves that were subsequently incorporated into the bone.

This interpretation is supported by XMT revelation of blood vessels crossing LAGs in canal-like waves and continuing along the length of the rib. Vascularization is predominantly longitudinal in distribution (see electronic supplementary material, movie S1). Bone deposition is increased and less organized on the visceral aspect of the rib (figure 3*f*,*g*), where vessels are more radially oriented (figure 3*d*,*e*,*f*,*g*).

The outer zone of bone is sharply demarcated. Its base is dark in transmitted and milky in polarized light. However, this may be an artefact caused by an uneven thickness of the section. Its external section exhibits stratification in transmitted light, which is inconspicuous on polarization. Radial, diverging canals extend to the surface. Older LAGs contain several well-vascularized, bleb-like protrusions of comparable sizes (much larger than typical, vascular canal-scale LAG waves) and location to those present on the surface. These were subsequently covered by younger zones of less-vascularized bone (figure 2; electronic supplementary material, movie S2). This suggests a chronic disease, spanning several seasons.

## 4. Discussion

### 4.1. Differential diagnosis

The observed blebs are typical of those occasionally seen in humans with *Mycobacterium tuberculosis*-derived tuberculosis [45,46]. XMT examination documents the blebs as solid (branching channels excepted), ruling out taphonomic blister formation and that related to vitamin C deficiency (scurvy). The periosteum is separated from the underlying cortical bone in scurvy [2,47]. Potential taphonomic explanation of branching channels is essentially limited to fungal disease, excluded because of size discrepancy. The diameter of fungal hyphae is five times larger [48]. Branching channels of a size similar to that of the vascular rib channels are, however, rarely noted with the branching bacterium *Actinomyces* [2].

There is no bone deformation, indicative of a healed fracture. Localization of the pathology to the internal (visceral) rib surface rules out a traumatic explanation. While developmental, dysplastic (e.g. fibrous dysplasia) and metabolic disorders (e.g. renal osteodystrophy) can involve ribs, their effect is limited to the internal bone structure. The periosteum is not altered, in the absence of subjacent bone involvement [2,39]. One exception is melorheostosis, a phenomenon in which ‘drip-patterns’ of periosteal reaction may occur without apparent underlying bone involvement [39]. However, that is macroscopically recognized by continuous dispersal down long bones, quite different from the focal spots (blebs) observed in MG UWr. 4438s. Pathology in the latter is clearly distinguishable from that of hypertrophic osteoarthropathy, in which periosteal reaction is present on the distal diaphyses of the appendicular skeleton [49]. These were apparently unaffected in MG UWr. 4438s. Rib involvement by cancer is typically related to metastatic disease directly invading the bone [2,39,50]. No such evidence was found in MG UWr. 4438s.

Primary bone tumours (e.g. osteoid osteoma) only secondarily affect the periosteum, and are recognized because of internal bone structure modification [39]. Gardner's syndrome, caused by a dominantly inherited mutation in the adenomatous polyposis coli tumour suppressor gene, may give a superficially similar osteological morphology [51]. Additionally, bone involvement in Gardner's syndrome is in the form of osteomas. They are spherical protuberances and do not have the flattened teardrop shape observed in our specimen. This is also true for button osteomas, which are also rounded, not tear-shaped. Further, such osteomas consist of Haversian bone [52], not the lamellar bone noted in MG UWr. 4438s. Other conditions that can produce periosteal proliferation are associated with osteolytic or osteoblastic internal bone reaction, absent in MG UWr. 4438s.

### 4.2. Rationale for suggesting tuberculosis as an inferred diagnosis for MG UWr. 4438s

The multidisciplinary approach used in the current analysis revealed a chronic, multi-seasonal disease affecting bone tissue. The presence of bone reaction and protrusions, localized to the visceral surface of the paravertebral area of the ribs, suggest a TB-type pulmonary infection, the oldest known such observation to date.

TB is well represented as a trans-phylogenetic disorder. All previously identified causative organisms have been members of the genus *Mycobacterium*, with species-dependent (phylogenetic) host specificity [23]. Most infections in humans are caused by *Mycobacterium tuberculosis.* Other mycobacterial infections (e.g. that caused by *M. kansasii*, *M. avium-intracellularis* and *M. thamnopheos*) have been recognized in extant animals, reptiles included [22–24]. Representatives of the genus *Mycobacterium* exhibit high tolerance to increased water salinity [53]. The genus is therefore believed to have emerged in the remote past as a primarily saprophytic, non-pathogenic [54] bacterium. It apparently acquired its virulence (pathogenicity) during long-term interactions with poikilothermic and subsequently with homoeothermic animals, given its wide taxonomic distribution [22,25].

The bleb-like periosteal bone apposition recognized in MG UWr. 4438s has been previously reported in humans with TB [45,46]. However, bone pathology is uncommon in humans (found in 1–2% of individuals with pulmonary disease) and blebs are even rarer. No other individual among the 10 000 animals examined to date by one of us (B.M.R.) has been found with such blebs (see also [2–4]). It is unclear why they have been so infrequently recognized. The mechanism of this particular morphology remains unknown. They may represent an unusual bone response in an immunosuppressed individual or even an aspect of disease pathophysiology (developmental course) that is so evanescent as to escape notice. It is apparent from the XMT scans of MG UWr. 4438s that the blebs developed during cycles of bone apposition. They were initially bordered by consecutive LAGs and eventually overlaid by newly deposited bone tissue during the next cycle, during which new blebs formed in neighbouring areas (electronic supplementary material, movie S2). This apparently was the response to local inflammation at the interface between the bone and surrounding tissues, possibly related to irritation by bacterial cells or their products. There is no clear evidence of gradual migration between spots. However, each bleb ends with a LAG. The bleb-inducing factor may have migrated, but no continuous record of such movement is preserved in the bone tissue, due to the cyclical arrest of bone apposition. Alternatively, the inflammation foci might have been stationary, appearing and disappearing in various locations during each cycle of bone growth. Dependence on some inducing factor, and the ability to resume normal bone growth in the area previously occupied by a bleb, allows consideration of the possibility that the blebs may be present only temporarily through the disease course, leaving no external traces. In animals (including many mammals) lacking or rarely developing LAGs or those with intensive bone remodelling, bleb ‘history’ may not be assessable. Nonetheless, determination of the occurrence and prevalence of blebs in the general zoological or anthropological record is beyond the scope of this study and will be monitored in a prospective manner.

Typical TB-related vertebral modification, referred to as Pott's disease [55], seems present in MG UWr. 4438s (see the electronic supplementary material). Although the specimen described herein is much too old to permit molecular-based (e.g. DNA) analysis, previous studies document that morphological clues may be successfully used to diagnose TB in a trans-phylogenetic manner and through geologic time [35]. Mycobacteriosis is common in modern reptiles [22], fish [56,57] and aquatic tetrapods [25–27], related to facile transmission of *Mycobacteria* spp. in aquatic medium [22]. Virulence of an ancient member of the *Mycobacterium tuberculosis* complex or of some other closely related fossil epizootic bacterial taxon developed much earlier than previously considered.

Many pathogens transfer horizontally across species, acquiring the ability during their evolution to colonize new groups of hosts or vectors. New hosts are sometimes acquired, independent of their evolutionary history and phylogenetic relationships. They originate as opportunists, infecting any taxa possible, only later specializing to infection or infestation of specific hosts. The susceptibility of those animals, whether pathologically affected or simply serving as vectors for transmission to disease-susceptible animals, is determined by initial (from an evolutionary perspective): (i) deficits of immunity against the given pathogen; (ii) physiological nuances (e.g. basal resting temperature); (iii) ecologically driven exposition; or (iv) newly acquired pathogen virulence [58,59]. This is illustrated by the distribution of modern pathogens, which is often spotty when it comes to taxa within families, orders or classes, but at the same time may involve more than one class. Therefore, the presence of infectious diseases in extant taxa does not necessarily indicate that their ancestors were similarly afflicted.

### 4.3. The impact of infection on the life of ‘*Proneusticosaurus*’ *silesiacus*

Given uncertainty as to whether the early sauropterygians lived in herds or exhibited any other gregarious behaviour, it is difficult to postulate the full epidemiologic potential of TB.

Modern-day epizootic studies of the spread of mycobacteriosis in free-ranging animals have been reported for fish [60,61], birds [62] and mammals [63], but data regarding reptiles are scarce. Lack of good sauropterygian analogues in modern ecosystems compromises further phylogenetic analysis at this time. However, another analytic pathway derives from consideration of ecotrophism. As the semi-aquatic lifestyle of nothosaurs is often compared to that of modern-day seals, it may be hypothesized that the character of disease transmission might have been comparable. Ecotrophism, an often overlooked aspect of organismal behaviour and spread of infectious disease, should be considered. Pinnipeds appear to be the marine mammal group most susceptible to TB [64]. Analogously, the first case of a TB-like disease is similarly found in a resident of a marine habitat.

Contrasted with extensive study of nothosaurid long bones [65–68], rib histology has received less attention [65]. Histological section of the MG UWr. 4438s rib reveals dense, very compact bone with a small medullary area (figure 3*a*,*b*). Such conditions seem to be consistent with that observed in the earliest (Early Anisian) representatives of *Nothosaurus* spp., characterized by increased bone mass. The latter is an adaptation, facilitating diving behaviour [65]. The ‘*Proneusticosaurus*’ *silesiacus* holotype is also characterized by relatively broad gastralia and unusually broad ribs (e.g. electronic supplementary material, figure S2), classified as pachyostotic [69]. Such adaptations are known in the secondarily aquatic tetrapods inhabiting shallow waters. They provide extra body mass, to compensate the positive buoyancy caused by atmospheric air accumulated in the lungs [70]. Contrasted with the physiology of open marine animals that are able to accumulate oxygen in the body tissues [71], the lungs in these primitive secondarily aquatic tetrapods are the main oxygen reservoir [71]. As pulmonary TB reduces respiratory function [72], breathing capacity was probably compromised in MG UWr. 4438s. This potentially constrained diving behaviour related to feeding or escape from predators. Decompression syndrome-related avascular necrosis, however, is relatively rare in nothosaurids [12,73]. Limited lung capacity might not have been detrimental for nothosaurids, if they were not routinely deep divers. While the part of the slab with the complete hand of MG UWr. 4438s is now missing, documentation provided by Volz [40] revealed neither hyperphalangy nor polydactyly. It had a typical nothosaurian autopodium structure, indicative of semi-aquatic adaptation.

It is clear that MG UWr. 4438s survived several seasons of disease, before it perished. It cannot be determined whether the death of MG UWr. 4438s was an independent phenomenon or the result of compromise of hunting efforts due to limited mobility related to diminished lung capacity or the result of TB-related failure of internal organs.

## 5. Conclusion

The case presented here is the oldest record of pneumonia and possibly pushes the earliest record of mycobacteriosis back to the Early Mesozoic, the dawn of the age of reptiles. Although the palaeopathological studies described herein are restricted in terms of available material and no typical microbiological or biochemical clues are accessible at this time, our diagnosis is supported by several lines of evidence—external and internal morphology of the blebs on the ribs, their distribution in the rib cage and the presence of vertebral damage comparable to that observed in Pott's disease. This is the first evidence of these in fossil reptiles and in such a distant past. The presence of probable TB-related changes on a skeleton of an extinct reptile living before the ascent of mammals supports the view of an ancient origin of mycobacterioses and their long-lasting influence on various vertebrate taxa.

## Supplementary Material

Supplementary Materials and Figures

## Data accessibility

All data are included in the paper and electronic supplementary material. Video files are available from the Dryad Digital Repository (https://doi.org/10.5061/dryad.59rm087).
