# Supplementary material for: Tuberculosis-like respiratory infection in 245-million-year-old marine reptile suggested by bone pathologies
Source: R Soc Open Sci. 2018 Jun 6;5(6):180225. doi: 10.1098/rsos.180225 (PMC6030318; doi:10.1098/rsos.180225)
Supplement: Supplementary Materials and Figures [file rsos180225supp1.docx]

**Tuberculosis-like respiratory infection in 245-million-year-old marine reptile suggested by bone pathologies**

**Supplementary information:**

**extended methods, specimen description, photographs
and CT scans**

**Dawid Surmik, Tomasz Szczygielski, Katarzyna Janiszewska, Bruce M. Rothschild**

**correspondence to: dawid@surmik.pl**

Content

[Extended methods – computed tomography (CT) 3](#_Toc498533582)

[Description of the specimen MG UWr. 4438s 3](#_Toc498533583)

[Pathology of vertebrae 5](#_Toc498533584)

[Supplementary Figure S1 7](#_Toc498533585)

[Supplementary Figure S2 8](#_Toc498533586)

[Supplementary Figure S3 9](#_Toc498533587)

[Supplementary Figure S4 10](#_Toc498533588)

[Supplementary Figure S5 11](#_Toc498533589)

[Supplementary Figure S6 12](#_Toc498533590)

[Supplementary Figure S7 13](#_Toc498533591)

[Supplementary Movie S1 13](#_Toc498533592)

[Supplementary Movie S2 13](#_Toc498533593)

[Supplementary Movie S3 13](#_Toc498533594)

[References 14](#_Toc498533595)

# Extended methods – computed tomography (CT)

CT examination of the whole specimen MG UWr. 4438s was performed with the GE Healthcare Discovery CT750 HD 64-channel computed X-ray tomography unit at the Department of Diagnostic Imaging of Regional Hospital of Trauma Surgery (Piekary Śląskie, Poland). CT scans with slice thickness of 0.625 mm were recorded as DICOM image files and processed and analyzed using GE Healthcare AW VolumeShare software and ImageJ [S1] (National Health Institute, USA).

# Description of specimen MG UWr. 4438s

The rib and vertebrae pathology was recognized by the present authors on the holotype (the only known specimen) of ‘*Proneusticosaurus*’ *silesiacus* (MG UWr. 4438s). Similar lesions were not found in other marine reptile skeletal remains from the Middle Triassic of Poland. ‘*P*.’ *silesiacus* is an enigmatic eosauropterygian genus known from two partially preserved postcranial skeletons from Upper Silesia, Poland. In 1902, Wilhelm Volz [S2] described two limestone slabs with trunk portions of skeletons from the early Middle Triassic (Lower Muschelkalk) of Gogolin and Zakrzów localities. Volz [S2] established two different species: ‘*Proneusticosaurus*’ *silesiacus*, for the remains from Gogolin, and ‘*Proneusticosaurus*’ *madelungii*, for the remains from Zakrzów. ‘*Proneusticosaurus*’ is considered by several authors [S3,S4] a junior synonym of *Cymatosaurus*, a coeval marine reptile genus which occurred in German Basin epicontinental sea [S5]. However, *Cymatosaurus* is described only from cranial material, and ‘*Proneusticosaurus*’ is represented only by postcranial remains, so no comparisons are possible. Both cranial and postcranial characters distinguish these taxa from the skull and skeleton characters of *Nothosaurus* [S6].

Originally illustrated by Volz [S2] (plate 15 therein), ‘*Proneusticosaurus*’ *silesiacus* was represented by a partially preserved skeleton embedded in a limestone slab, its ventral aspect exposed. The skeleton (figure 1*a* in the main text) is mostly articulated. Initial description identified presence of numerous thoracic ribs and densely packed gastralia, several dorsal vertebrae, nearly complete left forearms, part of the right radius, a complete right manus, nearly complete left manus (excluding carpals), nearly complete pelvic girdle with pubes and ischia in anatomical position, dislocated sacral vertebrae, sacral ribs, and a complete right femur with relatively well-developed trochanter. The specimen was housed in the University of Wroclaw, where it was substantially damaged during World War II. Only the middle portion survived, consisting of posterior dorsal ribs and gastralia, remnants of two digits of the left manus, several dorsal vertebrae, the complete right pubis, the medial part of the left pubis, the acetabular part of the right ischium and the proximal right femur (figure S1). There are distal fragments of at least six dorsal ribs visible on the right side of the animal (exposed in ventro-cranial view) and proximal or middle parts of at least five dorsal ribs on the left side (exposed in ventral view). The most proximal parts are missing and distal parts, either missing or obscured by gastralia and matrix. The exact sequential identity of these ribs cannot be established due to the incompleteness of the rib cage and disarticulation or lack of corresponding vertebrae. The ventral or dorsal surfaces of several vertebrae are exposed. CT scans of the whole MG UWr. 4438s slab reveal other skeletal parts embedded in the slab (figure S2), including pachyostotic ribs and vertebrae.

# Pathology of vertebrae

Less pronounced, but otherwise morphologically identical blebs are also present on the ventral and lateral surfaces of several exposed ribs and vertebrae (supplementary figures S3-6). CT scanning of the specimen MG UWr. 4438s (supplementary figure S2, supplementary movie S3) reveals that at least one bleb-bearing vertebra exhibits several focal regions of internal destruction, visible in CT scans as dark spots inside the centrum (supplementary figure S2*c-d*). The vertebra, itself, shows no external sign of mechanical damage or crushing and its walls are not collapsed. Thus, the internal erosions cannot be explained by simple taphonomic or diagenetic processes. The vertebrae of ‘*Proneusticosaurus*’ *silesiacus* are described as weakly platycoelous [S4]. Radiolucent regions are present throughout most of the centrum and near both ends of the vertebra described herein (supplementary figure S2*c-d*). Therefore, they cannot be explained as an artefact related to a single concave articular surface. The intervertebral space is represented on the scans as a thin, darker (radiolucent) line between the vertebra and is thus distinguishable from the radiolucent spaces described above. Furthermore, the osteolucent areas are asymmetrically located within the centrum. It is therefore also unlikely that they represent non-pathological internal spaces, such as large medullary cavities. For that reason, we interpret them as pathological. Given the presence of blebs on the ribs and vertebrae in that specimen, the pathology is most likely the remnants of vertebral tuberculosis [S7,S8]. The presence of similar focal destructions in other vertebrae of that specimen is ambiguous at the level of resolution and contrast achieved with current CT scans. Several other vertebrae may be affected, but their involvement cannot be confidently verified.

Pott’s disease – anterior vertebral collapse and fusion [S7] – has not been reported in reptiles and is not mentioned as a diagnostic criterion for mycobacterial infections in reptiles by Mitchell [S9]. Pott’s disease is considered one of the most definitive indicators for diagnosis of tuberculosis in humans, but is actually only found in one percent of affected individuals and appears to be the least commonly reported manifestation of tuberculosis in the non-human record of the disease [S9–S12]. The case presented here is apparently the first documentation of vertebral tuberculosis in a fossil reptile. The affected vertebra exhibits focal internal destruction, but lacks the major shape deformation that is often present in advanced cases of Pott’s disease in humans. Absence of collapse may relate to the horizontal orientation of the vertebral column in ‘*Proneusticosaurus*’ *silesiacus* and its aquatic mode of life, factors, which limit weight bearing-strain. Alternatively, the animal died before the bone disease fully developed. Limited skeletal preservation (both natural and post-recovery destruction) precludes estimation of the disease’s physiological and behavioural impact.


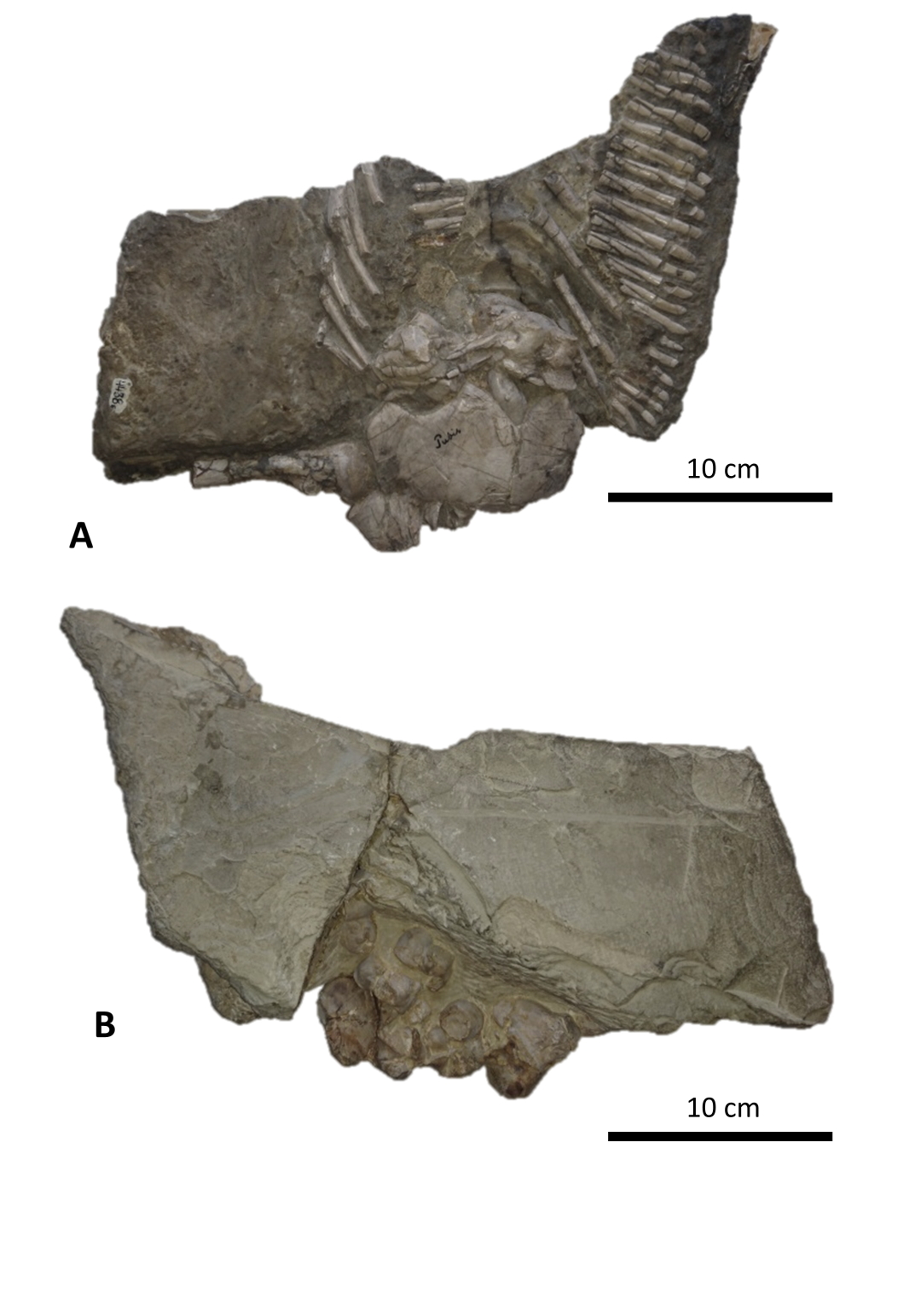


Supplementary Figure S1

*Proneusticosaurus silesiacus* holotype (MG UWr. 4438s) in ventral (A) and dorsal (B) aspect.


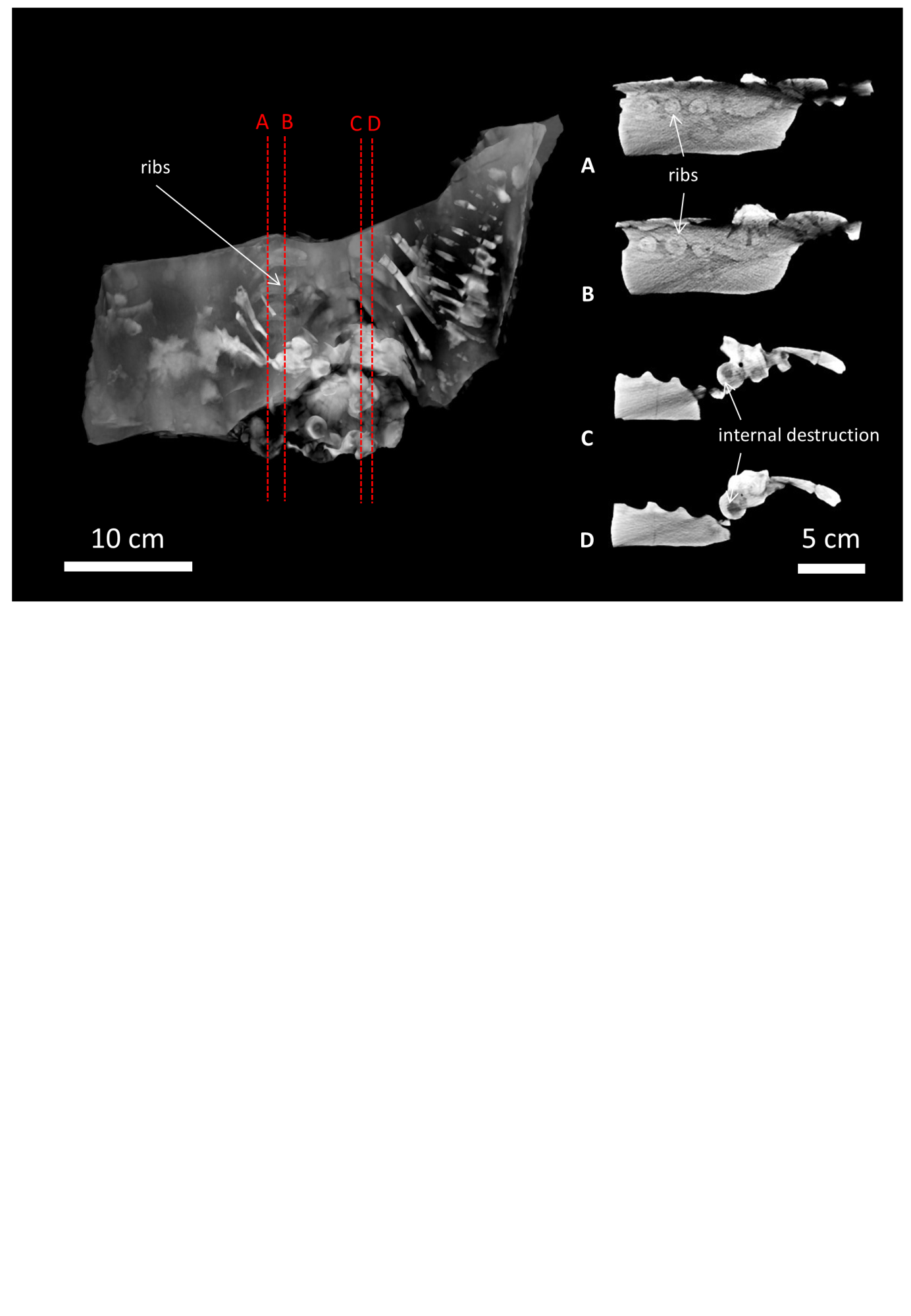


Supplementary Figure S2

Semi-transparent visualization of the slab of the specimen MG UWr. 4438s and selected CT sections, corresponding to particular slices (A–D). Note the presence of pachyostosis in dorsal ribs embedded in the slab.


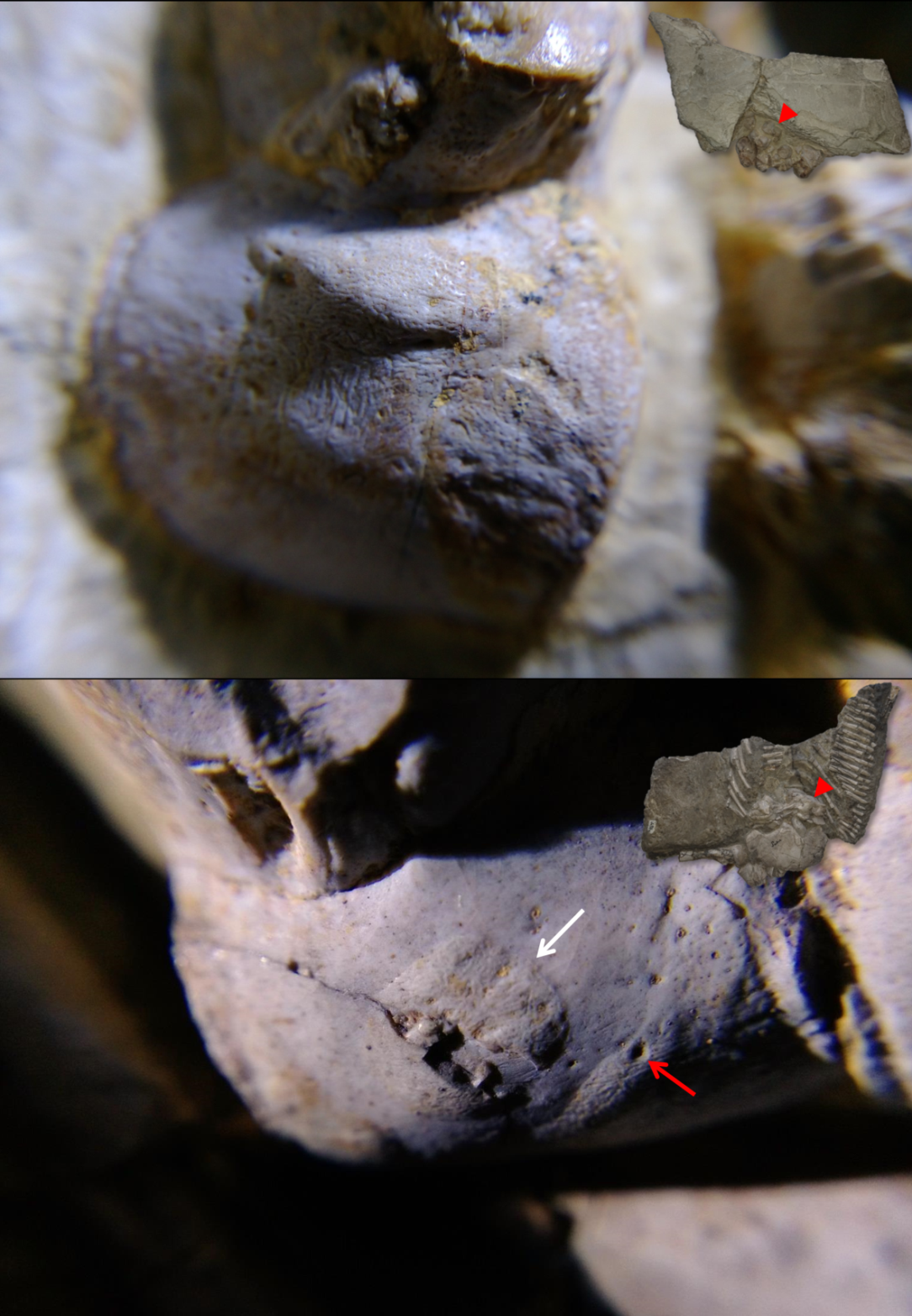


**B**

**A**

Supplementary Figure S3

Macrophotography of two of the exposed dorsal vertebrae in the dorsal (A) and ventral (B) part of specimen with protrusion (white arrows) and a vascular foramen (red arrow). Red pointers show the locations in the specimen. The vertebra in B contains pathological focal destruction within its centrum.


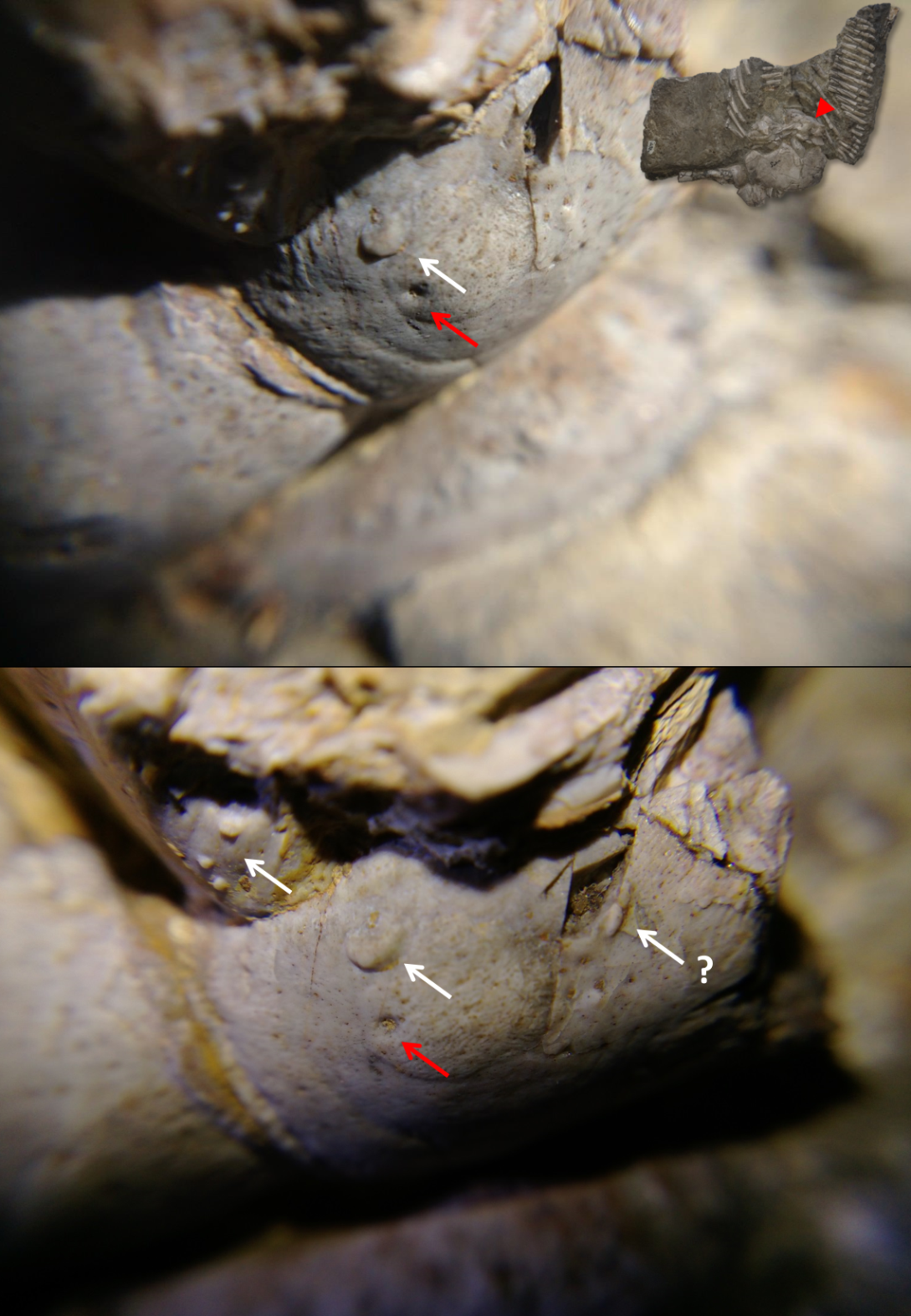


Supplementary Figure S4

Another exposed dorsal vertebra (adjacent to the one shown in Suppl. Fig. 2B) with weakly pronounced protrusions (white arrows) and a vascular foramen (red arrow) in varied lighting and at two different angles (not to scale). Red pointer shows the location in the specimen.


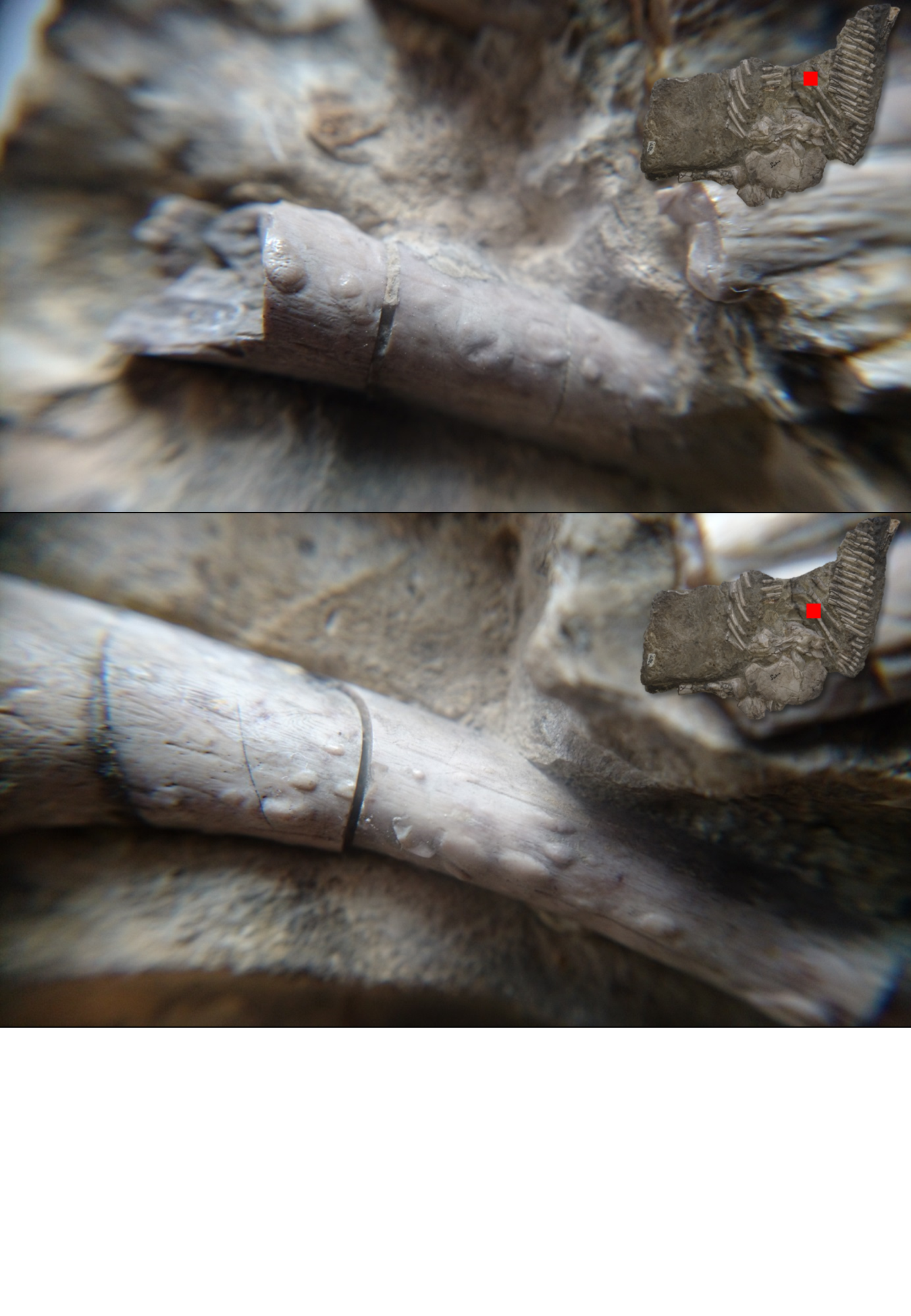


**A**

**B**

Supplementary Figure S5

Two of the exposed dorsal ribs with tear-shaped blebs on their visceral surfaces (not to scale). Red rectangles show the locations in the specimen.


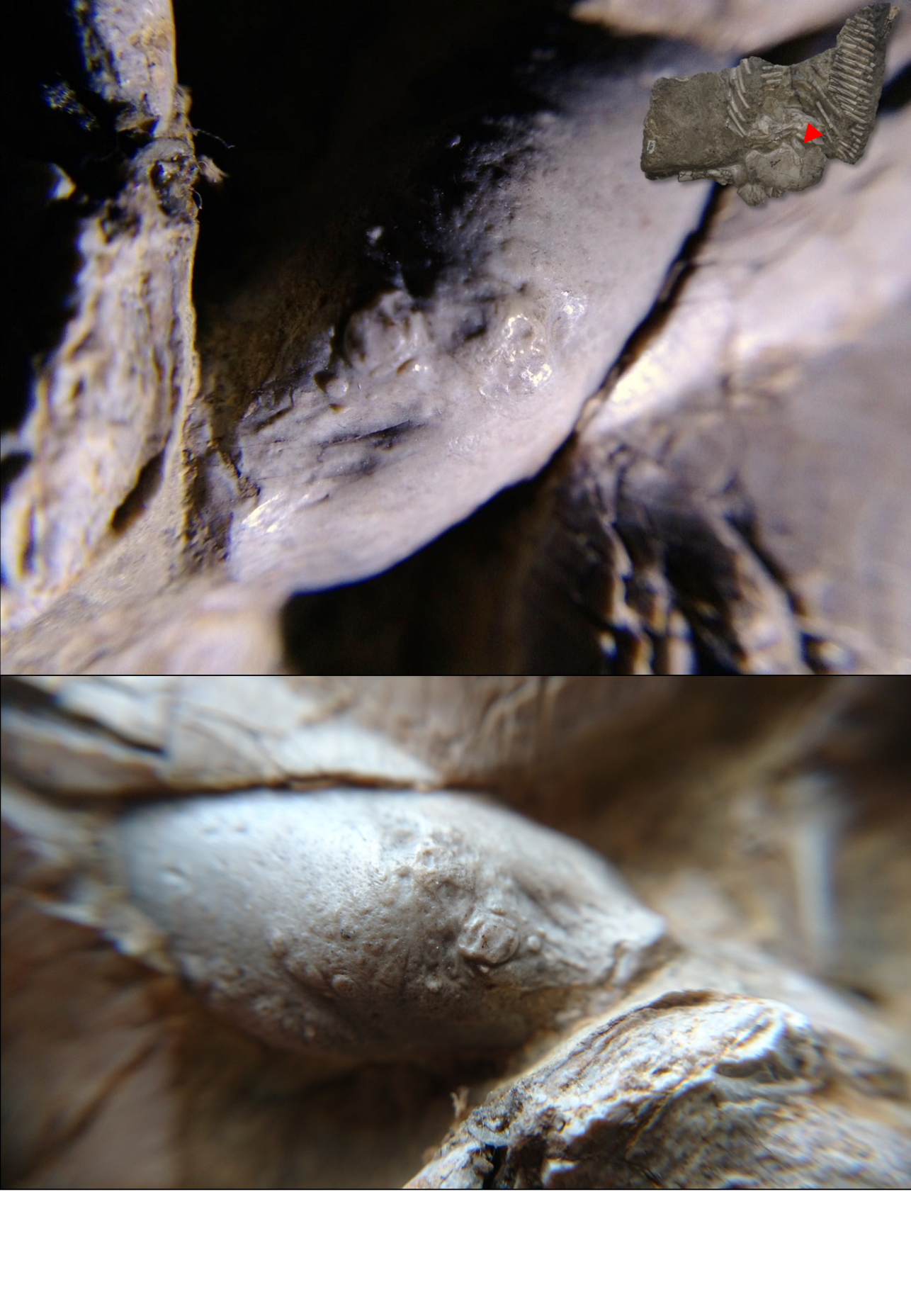


Supplementary Figure S6

Another rib fragment exposed in the pelvic region with similar protrusions shown in varied lighting and at two different angles (not to scale). Red pointer shows the location in the specimen.


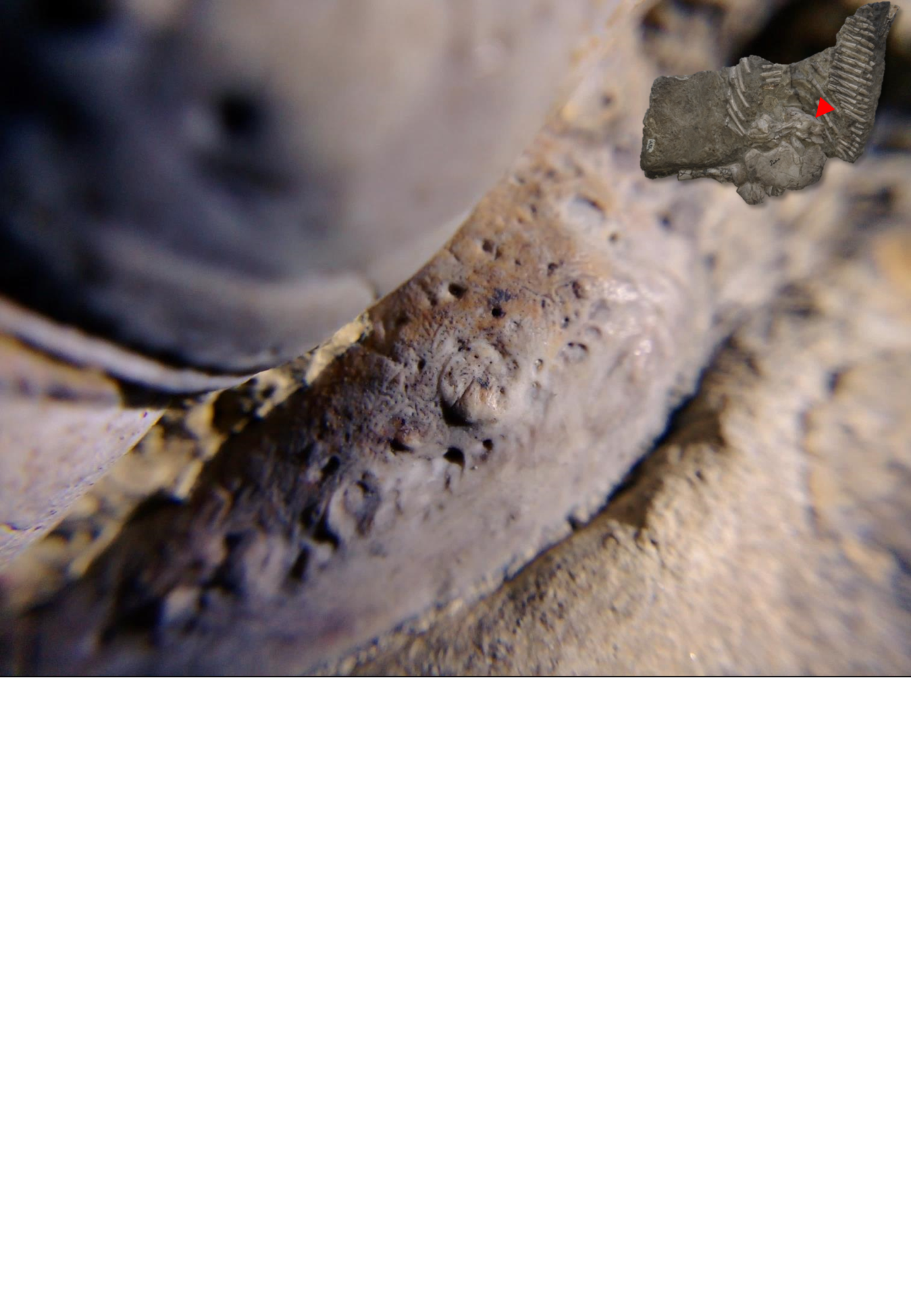


Supplementary Figure S7

**A**

A fragment of the rib covered by two pathologically-modified vertebrae shown in figs. S2B and S3, showing bleb-like protrusions. Red pointer shows the location in the specimen.

Supplementary Movie S1

Three-dimensional visualization of extracted rib fragment showing vasculature of the bone.

Supplementary Movie S2

XMT sections showing microstructure of rib.

Supplementary Movie S3

CT sectioning of whole specimen MG UWr. 4438s.

References

S1. Schindelin J, Rueden CT, Hiner MC, Eliceiri KW. 2015 The ImageJ ecosystem: An open platform for biomedical image analysis. *Mol. Reprod. Dev.* **82**, 518–529. (doi:10.1002/mrd.22489)

S2. Volz W. 1902 *Proneusticosaurus*, eine neue Sauropterygier-Gattung aus dem Unteren Muschelkalk Oberschlesiens. *Palaeontographica* **49**, 121–162.

S3. Sues H-D. 1987 Postcranial skeleton of *Pistosaurus* and interrelationships of the Sauropterygia (Diapsida). *Zool. J. Linn. Soc.* **90**, 109–131.

S4. Rieppel O, Hagdorn H. 1997 Chapter 5 - Paleobiogeography of Middle Triassic Sauropterygia in Central and Western Europe. In *Ancient Marine Reptiles* (eds JM Callaway, EL Nicholls), 121–144. San Diego Academic Press.

S5. Rieppel O. 1997 Revision of the Sauropterygian reptile genus *Cymatosaurus* v. Fritsch, 1894, and the relationships of Germanosaurus Nopcsa, 1928, from the Middle Triassic of Europe. *Fieldiana. Geology*, **36**, 1-21.

S6. Rieppel O. 2000 Handbook of Paleoherpetology / Sauropterygia I.: Placodontia, Pachypleurosauria, Nothosauroidea, Pistosauroidea: Part 12A. Stuttgart.

S7. Garg RK, Somvanshi DS. 2011 Spinal tuberculosis: A review. *J. Spinal Cord Med.* **34**, 440–454.

S8. Rothschild BM, Martin LD. 2006 *Skeletal impact of disease*. Albuquerque: New Mexico Museum of Natural History.

S9. Mitchell MA. 2012 Mycobacterial infections in reptiles. *Veterinary Clin. North Am. Exot. Anim. Pract.* **15**, 101–111. (doi:10.1016/j.cvex.2011.10.002)

S10. Zink AR, Grabner W, Reischl U, Wolf H, Nerlich AG. 2003 Molecular study on human tuberculosis in three geographically distinct and time delineated populations from ancient Egypt. *Epidemiol. Infect.* **130**, 239–249. (doi:10.1017/S0950268802008257)

S11. Anson C, Rothschild B, Naples V. 2012 Soft Tissue Contributions to Pseudopathology of Ribs. *Adv. Anthropol.* **02**, 57–63. (doi:10.4236/aa.2012.22007)

S12. Naples VL, Rothschild BM. 2011 Do ribs actually have a bare area? A new analysis. *HOMO - J. Comp. Hum. Biol.* **62**, 368–373. (doi:10.1016/j.jchb.2011.08.001)
